# Sociodemographic, Cognitive, and Emotional Determinants of Two Health Behaviors during SARS-CoV-2 Outbreak: An Online Study among French-Speaking Belgian Responders during the Spring Lockdown

**DOI:** 10.5334/pb.712

**Published:** 2021-02-22

**Authors:** Alix Bigot, Emilie Banse, Aline Cordonnier, Olivier Luminet

**Affiliations:** 1 UCLouvain, Research Institute for Psychological Sciences, BE; 2 Fund for Scientific Research (FRS-FNRS), BE

**Keywords:** pandemic, emotions, health behaviors, behavioral change, SARS-CoV-2, Theory of Planned Behavior

## Abstract

To contain the SARS-CoV-2 infection rate, health authorities have encouraged the population to enhance protective behaviors such as physical distancing and handwashing. Behavioral sciences emphasize the role of sociocognitive determinants to explain health behaviors, while largely ignoring emotional factors. In a large online study (N > 4000), we investigated the role of sociodemographic, cognitive, emotional, and social factors that can facilitate or hinder handwashing and limitation of social contacts. Data were collected from March 18 until April 19, 2020, which corresponds to the spring lockdown and the first peak of the pandemic in Belgium. Logistic regressions showed that sociodemographic factors (gender, age, level of education) and the dimensions of the Theory of Planned Behavior (intentions, attitudes, perceived behavioral control and subjective norms) had a strong impact on health behaviors, but that emotional factors explained an additional part of the variance. Being more *attentive/determined* and *frightened/anxious*, along with scoring higher on health anxiety were related to a higher frequency of handwashing. In contrast, being *enthusiastic/happy* was related to lower adherence to limiting social contacts. Our results suggest that the type of predictors and the direction of associations depend on the type of health behavior considered. The role of specific emotional factors in addition to more classical predictors is discussed. The study offers new perspectives regarding the factors that are associated with the adherence to behaviors recommended to adopt when faced with a pandemic.

The beginning of the year 2020 has been marked by the emergence of a new virus. SARS-CoV-2 spread across the world at such a high speed that it was soon referred to as a pandemic by the World Health Organization ([WHO], 2020a). Quickly, many governments introduced measures to prevent the virus from further spreading. The general goals of these measures were to reduce the number of social interactions and reinforce health behaviors, including handwashing (WHO, 2020b). But modifying the usual behavior of an entire population over the course of a few days is no easy feat. This is why it is important to better understand how different factors can facilitate or hinder the adoption of these behaviors. In this study, we aim to investigate the association of four categories of predictors (sociodemographic, cognitive, emotional and social) on the adherence to two central health behaviors recommended to face SARS-CoV-2: handwashing and limitation of social contacts. Those two behaviors can be distinguished on many aspects. Frequent handwashing is a classical preventive health behavior (WHO, 2020b). Most people already wash their hands frequently and understand its importance during pandemics (Gautier, Jauffret-Roustide & Jestin 2008). Washing hands is a behavior that is individually performed and under internal control, and can be more or less automatic depending on the person or the situation (Lunn et al., 2020). The second targeted behavior – limiting social contacts by staying home, avoiding public spaces and social distancing – is an avoidant behavior (Bish & Michie, 2010). Limiting social contacts has been a key measure to flatten the epidemic curve, yet it is difficult for people to inhibit their natural tendency to interact with others (Rigotti, De Cuyper & Sekiguchi, 2020). This recommendation requires to consciously stop a behavior that can be considered a habit (Gardner, 2015). However, habits rely on an automatic and nonconscious enactment of the behavior, making it difficult to change (Hagger et al., 2020). Furthermore, personal compliance with this behavior also depends on other people’s own compliance and might be modulated by social norms. It is also likely that additional factors can have divergent impacts on the two behaviors due to their distinct nature.

## Predictors of handwashing and limitation of social contacts

The principal aim of our study was to examine two particular types of predictors: sociocognitive predictors and emotional predictors. Sociocognitive predictors are often emphasized in theoretical models of behavioral change and in research assessing psycho-behavioral responses during pandemics (e.g., Bish & Michie, 2010). In our study, we included the four dimensions of the Theory of Planned Behavior (Ajzen, 1991; Conner & Norman, 2015) as sociocognitive predictors.

We also decided to consider the role of emotions. The SARS-CoV-2 pandemic created an unprecedented, sudden, unpredictable and highly novel situation that presented itself as an important threat to humanity (Van den Broucke, 2020). This particular context was thus very emotional for the whole population. Unfortunately, classical theories predicting health behaviors do not consider enough emotional determinants (Ferrer & Mendes, 2018). For this reason, we measured multiple different discrete emotions in order to capture the emotional states that might predict the two health behaviors specific to the SARS-CoV-2 pandemic.

In addition to cognitive and emotional components, we considered other categories of predictors. Sociodemographic dimensions (e.g., age, gender) were considered first in our analyses in order to evaluate the additional unique contribution of sociocognitive and emotional dimensions, over and above sociodemographic indicators. We also investigated other traits such as health anxiety, interoceptive sensibility and impulsiveness, and finally some dimensions relevant to the social context such as the degree of social relationships, empathy, and loneliness. The contribution of all these dimensions in predicting the two health behaviors assessed are detailed in the next section, in which we develop specific hypotheses for each of them. It is, however, important to note that the present study remains mainly exploratory due to the high novelty of the situation.

## Sociodemographic variables

### Age

Older individuals are more likely to carry out precautionary behaviors due to their higher risk perception (Barr et al., 2008; Bish & Michie, 2010).

### Gender

When gender differences are found, women are systematically more likely than men to implement protective behaviors during a pandemic (Bish & Michie, 2010; Leung et al., 2005). This could be explained by the caregiver and caretaker roles that women play in society, which place them more at risk of contracting the virus (WHO, 2007). In addition, women perceive themselves as more susceptible to contract an infectious disease (Gautier et al., 2008).

### Educational level

People who are more educated are more likely to adopt protective behaviors in case of a pandemic (for a review, see Bish & Michie, 2010).

### (Para)medical related work or studies

Healthcare workers are more at risk of contracting the virus, as they are more likely to be in contact with Covid patients. People who study in a field related to the (para)medical domain might also be more aware of the need to follow those measures, which should then be associated favorably with their application of health behaviors.

Overall, we hypothesize that sociodemographic variables will have a strong influence in predicting the two health behaviors, as suggested by the health literature.

## Theory of Planned Behavior (TPB)

The TPB (Ajzen, 1991) postulates that health behaviors can be directly predicted by our intentions to perform them. *Intentions* refer to the deliberate will to perform a behavior before applying it. They are, in turn, influenced by three sets of variables: attitudes, subjective norms and perceived behavioral control. *Attitudes*, which also include outcome efficacy, refer to the degree to which a person evaluates in a favorable or unfavorable way the behavior in question, and whether the implemented behavior will lead to positive or negative outcomes. *Subjective norms* are the perceptions of incentives from members of the social group to accomplish a certain behavior. Finally, *perceived behavioral control* involves the perceived ease or difficulty to perform a certain behavior, which refers to the construct of self-efficacy (Conner & Sparks, 2015). We hypothesize that the four components of the TPB will predict adherence to handwashing and limiting social contacts. Several meta-analyses have provided evidence for the ability of the components of TPB to predict general health behaviors (e.g., Conner & Norman, 2015), but also to predict precautionary behaviors in a pandemic context, such as willingness to limit social contacts (Zhang et al., 2019) or wearing a facemask (Chung et al., 2018).

## Current emotional states

Previous studies have already provided evidence that emotional variables predict behavior over and beyond the dimensions of the TPB, making the overall predictive model stronger (e.g., Conner et al., 2013). However, most of these studies only examined the affective states people *anticipate* they will be in when focusing on the future behavior to accomplish (Rivis, Sheeran & Armitage, 2009). Here, our goal is rather to include *current* emotional states. We consider that, in the context of the pandemic, an assessment of current emotional states was more relevant than the classical approach that focuses on anticipatory emotions (e.g., Ellis et al., 2018). Anticipated affect was not relevant as people were all currently experiencing sometimes strong affective reactions in response to what they were experiencing at the moment they completed the questionnaire.

In addition, there is an important gap in the literature concerning how specific affective states contribute to health decision-making and behavior (Ferrer & Mendes, 2018). Although there is an increasing number of studies examining the role of specific emotions and mood on decision making, there is still uncertainty regarding how emotions influence health behaviors (Lerner et al., 2015). So far, research has mostly focused on stress rather than emotional states (Ferrer & Mendes, 2018) and when they have considered the role of discrete emotional states, it has usually been limited to the specific role of fear (Witte & Allen, 2000).

While fear is a major affective response during pandemics (Van Bavel et al., 2020), several studies have also found that greater level of anxiety during a pandemic crisis predicted engagement in recommended behaviors, such as washing hands or covering one’s mouth when coughing or sneezing (Leung et al., 2003; Leung et al., 2005; Rubin et al., 2009). On the other hand, too much anxiety can also lead to cognitive avoidance strategies, which can minimize the perceived threat and therefore the adoption of the necessary behaviors (Van den Broucke, 2020). Negative emotions such as fear or anxiety can influence preventive behaviors through several mechanisms, including risk perception (Lunn et al., 2020; Van den Broucke, 2020), direct motivation (Turner & Underhill, 2012) or action tendencies generated by discrete emotions (Lazarus, 1991). These findings suggest that some negative emotions could play a central role in predicting the two behaviors we are interested in.

Positive emotions seem to favor adherence to healthy behaviors by increasing recommended actions and inhibiting risky ones (Lyubomirsky, King & Diener, 2005), although very few empirical studies have assessed the effects of positive emotions on health behaviors (Emerson, Dunsiger & Williams, 2017; Ferrer & Mendes, 2018) and none to our knowledge exist in the context of pandemics. Nevertheless, some theoretical frameworks can shed light as to how positive affects facilitate long-term adherence to health behaviors like the *broaden-and-build theory* (Fredrickson, 2013) or the *upward spiral theory of lifestyle change* (Van Cappellen et al., 2018). Consequently, one important contribution of this paper is to examine how different categories of negative as well as positive emotions can predict the adoption of the two considered behaviors.

## Health anxiety

Health anxiety – an “anxious preoccupation with the possibility that one has, or may have, a serious (usually fatal) disease, based on a misinterpretation of somatic symptoms” (Johnstone, 2010, p.723) – is another relevant dimension to consider (Jungmann & Witthöft, 2020; Mertens et al., 2020). We regard this dimension as a stable trait, as opposed to the emotional states listed above (such as fear or anxiety), which we introduced as measures of an intermittent state. We predict that people with low levels of health anxiety will be less prone to change social behavior or maintain social distances (Asmundson & Taylor, 2020).

## Interoception

Interoception refers to the processing of internal bodily signals by the nervous system (Khalsa et al., 2018). The interpretation that one makes of their internal bodily sensations plays a central role in physical and mental health, but also in various psychological phenomena (e.g., decision-making; Ceunen, Vlaeyen & Van Diest, 2016). As a result, better interoceptive abilities facilitate the assessment of one’s own health status, which increases the chances to act on it if necessary. We hypothesize that high levels of interoception will increase adherence to health behaviors.

## Impulsivity

Impulsivity is a multidimensional personality trait that includes lack of premeditation, urgency, sensation seeking and lack of perseverance (Whiteside et al., 2005). Limiting social contacts requires inhibitory processes that are deficient for highly impulsive individuals (Gardner, 2015). Therefore, we posit that high levels of impulsivity will reduce the application of limiting social contacts.

## Social connection

Measures of social connection include assessment of strength of social relationships, empathy, and feeling of loneliness. These measures differ from the social norms of the TPB as they reflect the level of social connection the individual currently has, whereas the social norms are more indicative of perceived social pressure. We hypothesize that, in the specific context of a lockdown where individuals are encouraged to inhibit their natural tendency to socialize, those three additional measures will provide complementary information regarding the limitation of the social contacts. One’s social network has a significant influence on behavior change, as it could spread positive health behaviors to a wide range of people (Kim et al., 2015). Moreover, in agreement with a recent study that showed that empathy promoted physical distancing during the SARS-CoV-2 pandemic (Pfattheicher et al., 2020), we expect that greater empathy will increase adherence to health behaviors as it fosters altruism and protection of others. Finally, as high levels of loneliness are associated with poor health (Segrin & Passalacqua, 2010), we anticipate a negative association between loneliness and adherence to the recommended behaviors.

## Method

### Participants

Participants were recruited on the internet, through posts on social networks, advertisements on university pages and emails. Furthermore, classical media (newspapers, radio stations, …) were also contacted. The study was presented as investigating the behaviors implemented to face SARS-CoV-2. The aim was to target a population as varied as possible regarding age, gender, education or work. However, they all had to speak French, as it was the language used in the questionnaire. We included in the analyses only participants who lived in Belgium. All participants provided their informed consent at the beginning of the study.

A total of 4853 respondents completed our questionnaire. Three participants were removed from the analysis because of aberrant data. We then excluded participants who responded “does not apply” on the item assessing the degree of agreement regarding the limitation of social contact, on the TPB’s items and on the ones corresponding to the social relationships, empathy and loneliness categories. However, to prevent a major loss of data, we decided to work with two separated samples for our two behaviors. Within each subsample, we kept participants who provided an answer other than “not relevant to me” on each item related to that particular behavior. Consequently, we obtained two groups: one examining sufficient handwashing (HW group, N = 4012) and one examining limitation of social contacts (LSC group, N = 4074).

In each group, the mean age of participants was around 42 years of age (ranging from 14 to 91, SD = 16.84 for HW group, SD = 16.79 for LSC group) and a high majority of respondents were females (around 75%). Approximatively 75% had a higher level of education, and around 27% were studying or practicing in a medical or paramedical field. Items assessing the presence of chronic disease revealed that around 17% of respondents, and just over 27% of close others were affected. Almost 10% stated being infected by SARS-CoV-2, with or without having been tested. Additionally, around 25% reported that one of several of their close family and friends had been infected. The items evaluating infection of coronavirus as well as suffering from a chronic disease were added three days after the study was launched. Because of the low response rate, these variables were not included in the analyses predicting the health behaviors.

### Procedure

The questionnaire was accessible through a link to a Qualtrics survey. It was online for four weeks, from March 18 to April 19, 2020. This period corresponds to the first phase of the epidemic in Belgium. March 18^th^ was the first day of lockdown and April 19^th^, the peak of the pandemic in Belgium (Sciensano, 2020). After providing their informed consent, participants completed questions investigating the following domains: TPB, emotions, personality traits (interoception, health anxiety and impulsivity), social connection and adopted health behaviors. Sociodemographic questions were positioned at the end of the questionnaire. Afterwards, participants were asked whether they would agree to be contacted for a follow-up study. If they agreed, they created an individual code and provided their email address. Participants were then thanked for their participation.

### Measures

The complete list of items can be found in the supplementary material. Here, we provide examples of items for each category of variable.

#### Health behaviors

Two items assessed health behaviors. To measure handwashing, participants indicated how frequently they washed their hands on a scale from 1 (never) to 5 (more than 15 times a day). To measure limitation of social contacts, participants indicated their level of agreement on a scale from 1 (completely disagree) to 5 (completely agree) with the statement “I limit my social contacts”. For this behavior, we included the option to answer “not relevant to me” as this behavior might not be possible for all participants (for example, individuals might have decided to move in with friends for the duration of the lockdown).

#### Demographic information

Participants specified their age in years, gender (male, female or other), and level of education. This latter variable involved 3 levels (from 1 corresponding to primary school, 2 to secondary school and 3 corresponding to higher education). In addition, they indicated whether their jobs/studies are in a medical or paramedical field and if they thought they or a close other had been contaminated by SARS-CoV-2 with or without being tested. We also had two items asking if the participant himself or a close other suffered from a chronic disease.

#### Intentions, attitudes, social norms and perceived control

To assess the central dimensions of the TPB (Ajzen, 1991), we created four items corresponding to the four different subscales and applied them to the two targeted behaviors (handwashing and limitation of social contacts). Participants answered them on a scale from 1 to 5. They had the additional option to report that the item did not apply to them. The wording of each statement was inspired by Conner and Sparks (2015). The dimension of intention was assessed with the item “I am ready to do *X*” (*X* represents the targeted behavior). Attitudes were assessed with the item “I believe that doing *X* will limit spreading of the coronavirus”, providing a perceived outcome efficacy measure. Social norms were investigated with the item: “My relatives expect from me to do *X*”. Finally, perceived behavioral control was assessed as a self-efficacy variable, with the item: “For me, doing *X* is easy”.

#### Emotions

To assess the current emotional states of participants, we administered an adapted version of the French Positive and Negative Affect Scale – State version (PANAS; Gaudreau, Sanchez & Blondin, 2006; Watson, Clark & Tellegen, 1988). We modified the original version in order to only include emotions likely to be experienced during the pandemic. Twenty emotions in total were assessed (10 positive and 10 negative). We conducted a factor analysis to reduce emotional states to a limited number of factors. The analyses indicated a four-factor solution. The conceptualization of our categorization essentially relies on this statistical procedure. The particular combination of emotions that we obtained was thus most likely affected by the very unique context in which we conducted the study. We labeled the first factor as *attentive/determined* as it included the items strong, determined, active, attentive and considerate (*α* = 0.76); the second factor as *enthusiastic/happy* as it included the items enthusiastic, happy, exalted and proud (*α* = 0.67); the third factor as *anger/agitation* as it included the items annoyed, irritable, angry, agitated and sad (*α* = 0.78) and the fourth factor as *fear/anxiety* (*α* = 0.77) as it included the items distressed, guilty, scared, frightened, anxious and surprised.^1^

#### Health anxiety

As mentioned in the introduction, health anxiety is a distinct construct from fear. Empirical data support this assumption by showing a moderate correlation between this construct and our *fear/anxiety* factor (*r* = 0.45), suggesting that the two dimensions only partially overlap. To measure attitudes toward health, we selected items from the Whiteley Index (WI) developed by Pilowsky (1967). As it seems that no French-version of the questionnaire exists, we translated the Dutch-version of the WI (Speckens et al., 1996) into French using the back-translation method (Triandis, 1980). We then selected 5 items with strong face validity evaluating concerns about the threat of becoming sick (e.g., “I’m afraid of getting sick.”). The 5 items were averaged to make a single index of “health anxiety” (*α* = 0.69).

#### Impulsivity

This personality trait was evaluated with the French version of the UPPS impulsive behavior scale (Whiteside et al., 2005; Van der Linden et al., 2006). We selected the six items with the highest loadings on each factor of the French validation. Of those six items, two assessed the dimension premeditation (*α* = 0.65) and two the dimension urgency (*α* = 0.74). The last two single items represented the sensation seeking and perseverance dimensions.

#### Interoception

We administered ten items from the Three-domains Interoceptive Sensations Questionnaire [ThisQ]. This scale was developed by Vlemincx and colleagues (2020) to assess interoceptive sensibility by focusing on items without any emotional context. We selected five items from the respiratory domain, as this domain closely relates to SARS-CoV-2, and five from the cardiac domain, as it is the most common domain assessed in interoceptive sensibility. We ran a factor analysis with a two-factor extraction that corresponds to the factors obtained in Vlemincx et al. (2020), which are cardio-respiratory activation (*α* = 0.80), and cardio-respiratory deactivation (*α* = 0.82).

#### Social connection

We combined items from different sources to measure participants’ level of social connection. The first three items assessed current relationships between the participants and close others (family, friends and neighbors) on a scale from 1 (no bond) to 5 (very strong bonds; Morton et al., 2020). Afterwards, participants indicated their level of agreement with 11 items. Four items originated from the Individualism and Collectivism Scale (Triandis & Gelfand, 1998).^2^ Four items were selected from the Interpersonal Reactivity Index (IRI; Davis, 1983) and measured empathy. The 3 remaining items assessed loneliness and came from a Short Scale for Measuring Loneliness in Large Surveys (Hughes et al., 2004). Across this section, participants could respond by indicating that the sentence did not apply to them”. Factorial analyses extracted four components. We conceptualized them as representing *social relationships* (*α* = 0.59), *empathy* (*α* = 0.75) and *loneliness* (*α* = 0.83). The last one, labeled *individualism*, was not used due to its insufficient Cronbach’s alpha.^3^

In agreement with the assumption that these predictors would be complementary to TPB’s social norms, we found low correlations between all variables measuring social connection and social norms (*r*s ranging from 0.01 to 0.14). These data confirm the absence of overlap between the three social dimensions and the TPB’s.

### Data analyses

Parallel items assessing TPB’s dimensions were systematically compared through paired-sample t-tests in order to investigate the specific level of intentions, attitudes, social pressure or perceived behavioral control to each behavior. The same analysis was conducted to investigate the prevalence of our distinct categories of emotions, and observe their current importance reported by participants.

We used binary logistic regressions to assess the adherence to two important health behaviors in the context of SARS-CoV-2: sufficient handwashing (HW) and limitation of social contacts (LSC) using SPSS statistical software (version 26). The analyses were carried out on each behavior individually. Regarding sociodemographic information, we combined the two first levels of education (primary and secondary school) in order to obtain a number of participants proportionally equivalent to the number of higher educated participants. To create binary variables, we coded each recommended behavior as applied or not. For handwashing, participants had to indicate washing their hands more than 6 times a day. We set this level based on the recommendations established by several sanitary institutions which indicated that people should wash their hands at the very least before and after eating, and after using the bathroom. For social distancing, participants had to agree or totally agree with the statement “I limit my social contacts”. We then added other variables by block as potential predictors of each behavior. It is important to note that all predictors used in the two logistic regressions were identical, except items related to the TPB. Indeed, questions measuring the four scales of the TBP were specific to the studied behavior.

## Results

### Descriptive data

A total of 2850 out of 4012 (71%) participants sufficiently washed their hands and 3762 out of 4074 (92.3%) reported limiting their social contacts. It is worth noting that about 66% of participants included in both samples applied both recommended behaviors. Means and standard deviations of continuous predictors for each group (HW and LSC) can be found in ***Table 1***.

**Table 1:** Means and Standard deviations of Continuous Predictors.


	HANDWASHING (HW) N = 4012	LIMITATION OF SOCIAL CONTACTS (LSC) N = 4074

	MEAN (SD)	MEAN (SD)

Intention	4.65 (0.69)	3.93 (1.26)

Attitude	4.39 (0.82)	4.50 (0.84)

Subjective norms	3.97 (1.09)	4.04 (1.08)

Perceived control	4.39 (0.89)	2.11 (1.33)

Attentive/Determined	3.50 (0.75)	3.50 (0.75)

Enthusiastic/Happy	2.30 (0.80)	2.30 (0.80)

Angry/Agitated	2.78 (0.95)	2.79 (0.95)

Frightened/Anxious	2.53 (0.83)	2.54 (0.83)

Activation	3.95 (0.70)	3.95 (0.70)

Relaxation	3.75 (0.82)	3.75 (0.81)

Health anxiety	2.86 (0.78)	2.86 (0.78)

Premeditation	3.94 (0.72)	3.95 (0.72)

Urgency	2.51 (1.03)	2.51 (1.03)

Sensations seeking	2.09 (1.03)	2.08 (1.03)

Perseverance	4.02 (0.95)	4.01 (0.95)

Social relationships	3.41 (0.71)	3.41 (0.72)

Empathy	4.17 (0.60)	4.18 (0.59)

Loneliness	2.55 (1.16)	2.57 (1.16)


*Note*: Range between 1 and 5.

As emotions are central to our study, we started by comparing the prevalence of our four categories of emotions within participants from both samples combined (N = 3938) using paired-samples t-tests. Results revealed significant differences for each of our six comparisons (i.e., all categories were significantly different from one another). To assess the importance of these differences, we calculated Cohen’s *d* and based our interpretations on Cohen’s thresholds with values around 0.2 indicating small effects, 0.5 for medium effects, and around 0.8 for large effects (Cohen, 2013). Emotions relating to the category *attentive/determined* were felt more strongly (*M* = 3.50, *SD* = 0.75) than the other three categories of emotions (*enthusiastic/happy, angry/agitated* and *frightened/anxious*), with medium to large effect sizes (*d*s = 0.52 to 1.37). Only small differences between the other three categories of emotions were observed (Cohen’s *d* ranging from 0.19 to 0.36) as they were felt with medium emotional intensity by our participants (*M*s between 2.30 and 2.78, *SD*s between 0.75 and 0.94). Overall, participants felt highly *attentive/determined* during the SARS-CoV-2 outbreak, and they felt moderately *angry/agitated, frightened/anxious* and *enthusiastic/happy*, with only small differences in terms of intensity between these three emotional categories.

We also conducted paired-sample t-tests to compare each TPB’s dimensions (intentions, attitudes, subjective norms and perceived control) across the two health behaviors. Results showed that intentions to apply the behavior and the perceived control over it were significantly higher for handwashing than for limiting social contacts, with a medium effect size for intentions (*d* = 0.55) and a large effect size for perceived control (*d* = 1.46). In contrast, participants had a more positive attitude and felt more social pressure regarding their limitation of social contacts than their handwashing. However, the effect sizes were of small magnitude (*ds* = 0.10 and 0.04).

### Logistic regressions

Two binary logistic regressions were conducted to predict application or non-application of the investigated behaviors. Six different blocks of variables were included in the analysis (see ***Table 2***)^4^ with the ENTER method.^5^ We used the Nagelkerke *R^2^* in order to assess the collective importance of the predictors included in our model, relative to a “perfectly fitting” null model (Nagelkerke, 1991).^6^

**Table 2:** Logistic Regressions Analyses Associated with the Application of Recommended Behaviors.


	HANDWASHING (N = 4012)					LIMITATION OF SOCIAL CONTACTS (N = 4074)				
	
	B	EXP(B)	95% C.I. FOR EXP(B) LOWER–UPPER	*𝜒*^2^ BLOCK	NAGELKERKE’S *R*^2^	B	EXP(B)	95% C.I. FOR EXP(B) LOWER–UPPER	*𝜒*^2^ BLOCK	NAGELKERKE’S *R*^2^

BLOCK 1 – Sociodemographic aspects				161.06***	0.056				71.32***	0.042

Gender	0.40***	1.50	1.25–1.80			0.21	1.24	0.93–1.63		

Age	0.01***	1.01	1.01–1.02			–0.03***	0.97	0.97–0.98		

Level of education	0.07	1.08	0.90–1.29			0.53***	1.70	1.30–2.24		

(Para)medical field	0.32**	1.37	1.14–1.64			–0.04	0.97	0.72–1.29		

BLOCK 2 – TPB’s components				499.63***	0.217				115.89***	0.108

Intention	0.59***	1.81	1.59–2.05			0.14*	1.14	1.02–1.29		

Attitude	0.14**	1.15	1.03–1.27			0.32***	1.38	1.20–1.59		

Subjective norms	0.07	1.07	0.99–1.15			0.21**	1.23	1.09–1.39		

Perceived control	0.42***	1.52	1.39–1.67			–0.00	0.99	0.90–1.11		

BLOCK 3 – Emotions				43.73***	0.230				16.39**	0.117

Attentive/Determined	0.22**	1.24	1.10–1.41			0.16	1.18	0.97–1.43		

Enthusiastic/Happy	–0.06	0.94	0.84–1.05			–0.31**	0.73	0.61–0.87		

Angry/Agitated	0.11	1.12	0.99–1.25			–0.16	0.85	0.71–1.02		

Frightened/Anxious	0.15*	1.16	1.01–1.32			0.17	1.19	0.96–1.47		

BLOCK 4 – Physiological aspects				6.78	0.232				1.18	0.117

Health anxiety	0.12*	1.13	1.00–1.27			–0.10	0.91	0.75–1.09		

Intero-activ	0.09	1.09	0.96–1.23			–0.04	0.96	0.79–1.16		

Intero-relax	–0.00	0.99	0.90–1.11			0.06	1.06	0.90–1.26		

BLOCK 5 – Impulsivity				5.41	0.234				4.98	0.120

Premeditation	–0.02	0.98	0.87–1.10			0.02	1.02	0.85–1.22		

Urgency	–0.02	0.98	0.87–1.10			0.02	1.02	0.89–1.16		

Sensation seeking	–0.08	0.92	0.85–1.00			–0.00	0.99	0.87–1.14		

Perseverance	0.03	1.03	0.95–1.12			0.14*	1.15	1.02–1.31		

BLOCK 6 – Social connection				7.21	0.236				6.43	0.124

Social relationships	0.11	1.12	0.99–1.26			–0.21*	0.81	0.68–0.98		

Empathy	0.07	1.08	0.94–1.24			0.09	1.10	0.88–1.37		

Loneliness	–0.04	0.97	0.93–1.04			0.05	1.05	0.93–1.19		


*Notes*: B values reported in the table are the ones obtained at step 6. Intero-activ and Intero-relax refer to the activation and deactivation factors of interoception. Presented data were extracted after entering all blocks of predictors with the ENTER method. * p < 0.05, ** p < 0.01, and *** p < 0.001.

#### Sufficient handwashing

The full model, which includes all variables, was significantly related to sufficient handwashing in 76% of cases, with a *R^2^* of 23.6%; χ^2^(22, *N* = 4012) = 723.83, *p* < 0.001. The first block, which includes sociodemographic predictors, revealed a significant effect of gender, age and working or studying in the medical/paramedical field; χ^2^(4, *N* = 4012) = 161.06, *p* < 0.001. Being female, older, and practicing in the medical/paramedical field increased the likelihood of washing one’s hands often enough. Three out of the four TPB dimensions added in the second block of the analysis were also retained as significant predictors. Intentions, positive attitudes and a high perceived control over the capacity to apply the behavior predicted the adherence toward it. This block significantly increased the predictive power of the model; χ^2^(4, *N* = 4012) = 499.63, *p* < 0.001. The third block, which includes emotional variables, showed significant effects for the *attentive/determined* and *frightened/anxious* categories and increased the predictive power of the model; χ^2^(4, *N* = 4012) = 43.73, *p* < 0.001. High scores in these two categories of emotions were associated with a higher probability of applying the behavior. Although the next two blocks did not increase significantly the *R^2^*, a higher score on the index measuring anxiety toward health was related to higher likelihood of sufficient handwashing.

#### Limitation of social contacts

The full model significantly predicted limitation of social contacts; χ^2^(22, *N* = 4074) = 216.20, *p* < 0.001. Together, the six blocks of predictors explained 12.4% of the total variance, with a total of 92.3% participants correctly classified by the model. The first block of sociodemographic features revealed that older age was related to a decreased probability of limiting social contacts. In addition, having a high level of education increased the likelihood of following the recommended behavior. By themselves, sociodemographic variables significantly predicted the expected outcome; χ^2^(4, *N* = 4074) = 71.31, *p* < 0.001. The second block of predictors, which includes the four dimensions of the TPB, significantly increased the predictive power of the model; χ^2^(4, *N* = 4074) = 115.89, *p* < 0.001. All variables but perceived control over the behavior were significantly associated with a greater probability of applying the behavior. Results for the third block, which includes emotional states, indicated that a higher score on the *enthusiastic/happy* category was associated with a decreased probability to limit social contacts. This block also significantly increased the model’s power; χ^2^(4, *N* = 4074) = 16.39, *p* < 0.001. The following blocks did not significantly improve the model’s predictions. Nonetheless, perseverance score, which is one component of impulsivity, was significantly related to the dependent variable. A greater capacity to mobilize efforts despite difficulties was associated with a higher probability to respect social contacts limitation. Finally, in the last block about social connection aspects, results showed that a greater investment in social relationships was related to lower adherence to the behavior.

## Discussion

### Overview

This study sheds light on the distinct predictors that are associated with each behavior. Indeed, while the TPB and emotions blocks were both significant in each model, the variables that were related to handwashing were distinct from the ones associated with the limitation of social contacts. In this discussion, we address the differences in pattern by keeping in mind the mechanisms that underlie the two behaviors. Subsequently, we direct our focus on the role of emotions since their inclusion as health determinants was a major contribution to this study. We conclude by discussing the limits and implications of our results.

### Predicting sufficient handwashing and limitation of social contacts

When faced with a pandemic, it is essential to understand which factors can lead to the adherence to health behaviors. In this study, we used logistic regressions to examine how a broad range of variables were related to handwashing and limitation of social contacts in the context of the SARS-CoV-2 pandemic. First, sociodemographic variables predicted the two behaviors, although in different ways. On the one hand, being female, older, and working or studying in the (para)medical field increased the likelihood of washing one’s hands frequently. On the other hand, being younger and having a higher level of education increased the probability of limiting social contacts. TPB’s dimensions also differed across behaviors. Whereas higher intentions and more positive attitudes were related to higher adherence to the two behaviors, higher perceived control was only associated with more handwashing, while subjective norms were only related to the limitation of social contacts. As for emotions, specific effects were observed. Regarding frequency of handwashing, the application of the behavior was related with both being attentive/determined, and frightened/anxious. Interestingly, this behavior was also linked to a higher level of anxiety towards health. For the limitation of social contacts, participants with high scores on the *enthusiastic/happy* category were less likely to follow the recommended behavior. However, an alternative interpretation should be considered: individuals who did not limit their social contacts might feel more positive emotions as they are less confronted by isolation. Two additional effects were observed for the social contacts limitation. The application of the behavior was associated with high scores on perseverance and low scores on social relationships investment.

It is important to review these results with regard to the existing literature. We will do so by considering the different nature of the two behaviors investigated as they rely on different underlying mechanisms that were extensively developed in the introduction. Regarding sociodemographic aspects, the finding that older participants washed their hands more frequently is consistent with previous studies assessing behaviors during pandemics (e.g., Barr et al., 2008) and can be explained by their higher susceptibility to the disease, leading them to follow sanitary measures. However, older age was also related to less limitation of social contacts. In a recent study, Chan and colleagues (2020) found that being younger was associated with more anxiety of being infected by SARS-CoV2. We found similar results as younger participants obtained higher scores in the *fear/anxiety* factor, which could then lead to more limitation of social contacts. In addition, younger people are generally more sensitive to peer pressure (Knoll et al., 2017), which might also contribute to a better adherence to the limitation of the social contact rule.

The finding that women wash their hands more frequently is an often-observed gender difference (e.g., Borchgrevink, Cha & Kim, 2013). We hypothesize that this result can be explained by two factors: gender roles and risk perception. The roles women play in society often place them at higher risk of contracting the virus and thus, women would be likely to follow sanitary measures (WHO, 2007). Gender differences related to risk perception is another possible explanation. Recent studies related to SARS-CoV-2 (e.g., Dryhust et al., 2020) found that men perceived less risk than women and that greater risk perception was associated with greater adoption of preventive health behaviors such as handwashing. Paradoxically, this perception is not in line with actual risks for SARS-Cov-2 related mortality, which appear to be higher for men than for women.

Level of education was only significantly linked to limitation of social contacts with more educated people adhering to this behavior. This result is consistent with the idea that more educated people are better able to engage in health behavior, and that education has a positive influence on their adherence (Bish & Michie, 2010; Margolis, 2013). Finally, people who work or study in a (para)medical field washed their hands more frequently. This effect can be explained by higher risk exposure and higher threat perception (WHO, 2020c).

The TPB significantly increased the predictive power of the model for both behaviors, confirming that this theory includes important variables to predict changes in health behaviors, even in a context such as a pandemic. However, the two health behaviors targeted in this study were not significantly predicted by all components of the TPB. Intentions, the motivational aspects of a behavior, was significantly associated with both handwashing and limitation of social contacts, a result in contradiction with previous studies that failed to find a significant effect of intentions on precautionary behaviors during pandemics (e.g., Chung et al., 2018). Attitudes were also significantly related to both behaviors, which suggests that people who acknowledge and understand the importance of limiting social contacts and handwashing are more inclined to act accordingly. Indeed, perceived efficacy of a preventive behavior influences the motivation to accomplish that behavior (Van den Broucke, 2020). Regarding the two other important components of the TPB, contrasted effects were observed, pointing to the different nature of the two behaviors. Looking at the limitation of social contacts, descriptive statistics showed that participants stated having a low perceived behavioral control on this behavior. This suggests that this behavior is still new and unusual for people, whereas handwashing is a more usual behavior. Increasing one’s frequency of washing hands is largely associated with perceived behavioral control, attesting that this behavior is largely under internal control. In contrast, the motivations to limit social contacts are very different as it involves avoidance of prosocial behaviors and relies on inhibitory processes (Gardner, 2015). Our results show that limiting social contacts is not linked to behavioral control, but rather to subjective norms. This highlights the role of peers in accomplishing a behavior that would be very unusual in normal circumstances.

A central purpose of this study was to address the role of specific emotions in the prediction of health behavior as a complement to the TPB’s dimensions (Conner et al., 2013). Based on factorial analyses, we included four categories of affective processes in order to investigate their impact on health decisions and behaviors. This is coherent with Ferrer and Mendes (2018) who advocated for using discrete emotional states to predict health behaviors. Three of our categories of emotions were related to the behaviors. First, high *fear/anxiety* was related to sufficient handwashing. One explanation of this effect relates to the susceptibility and severity of the infection threat one can perceive when experiencing strong fear appeal. These negative emotions act as a protective mechanism that motivates adaptive danger control actions and provides resources to facilitate the change of behavior (Witte & Allen, 2000). However, the level of fear arousal is an important determinant. Intense levels of fear can lead to cognitive avoidance strategies by minimizing threat perception and decreasing adherence to health behaviors (Croyle, Sun & Hart, 2013). Hence, perceived threat is only effective if it is associated with a sense of control and strategies to face this emotion (Van den Broucke, 2020).

In addition to general *fear/anxiety*, results also showed that a more clinical emotional dimension – health anxiety (also called hypochondria) – was related to frequent handwashing. However, whereas general anxiety or fear during a health crisis such as the SARS-CoV-2 pandemic is generally adaptive, high health anxiety scores would most likely lead to dysfunctional behaviors. Jasper and Witthöft (2013) revealed the presence of several non-adaptive processes associated with health anxiety, such as rumination and catastrophizing. Furthermore, they also showed that a negative automatic evaluation of illness-related stimuli appears to be strongly involved in hypochondria. In summary, although both general anxiety and hypochondria were related to sufficient handwashing, further investigations should be conducted to distinguish the different processes specific to each of these predictors.

One final major contribution of this study is the inclusion of positive specific emotional states. Regarding the frequency of handwashing, we found that emotions akin to motivational aspects – attention and determination – were related to a greater adherence to the behavior. We propose an interpretation based on the *Upward Spiral Theory of Lifestyle Change* (Van Cappellen et al., 2018). This theoretical framework postulates that positive affects create new resources that encourage adaptive behaviors. Regarding the limitation of social contacts, we obtained an unexpected result. High levels of happiness were inversely associated with participants’ propensity to limit their social contacts. One interpretation relies on the fact that these emotions are characterized by high levels of arousal (Bodenhausen, 1993). Isen and colleagues (1982) showed that individuals rely more on heuristic response strategies when feeling happy, which can then yield erroneous judgments. Social interactions and more broadly feelings of belonging are natural behaviors and are normally considered sources of well-being. Hence, it is possible that people who feel strong positive emotions would be less willing to reduce their social interactions, in spite of the risks involved. Another explanation might rely on the fact that, when facing threat, individuals turn to social support to help them cope with adversity (Harlow & Cantor, 1995). Consecutively, lower social and emotional support obtained due to the decrease of social interactions during the lockdown might then be associated with lower positive emotional states, while keeping social contacts in a context of social isolation might contribute to enhance positive emotions. We found supporting evidence of these interpretations in our results. Individuals who indicated being highly invested in their social relationships were less likely to respect the recommended behavior. A final note regarding the emotional factors involved in the present study is that they somewhat differ from a classical arousal by valence structure. Indeed, in one of the two negative emotion factors, we found a mix of low (sad, annoyed) and high (irritable, angry) arousal states. Future studies should examine the stability of this structure in the context of the SARS-CoV-2 pandemic.

To conclude, limiting social contacts is a challenging recommendation as it involves the inhibition of a habitual behavior (Gardner, 2015). We found that individuals who demonstrate perseverance in the face of difficulties are more likely to respect the limitation of social contacts. Accordingly, impulsivity models show that the capacity to inhibit behaviors effortlessly is associated with better self-control (Galla & Duckworth, 2015). Hence, this process might facilitate the formation of health behaviors and overall adaptive habits.

### Implications of the study

Our study has highlighted important psychological processes to consider when trying to increase people’s adherence to health behaviors in contexts such as the SARS-CoV-2 pandemic. Several interventions can then be envisioned in order to modify health behaviors. An interesting tool to augment adherence to handwashing would be to use *nudging*, a technique that provides material resources in order to ease the application of a specific behavior. For example, handwashing could be increased by placing hand-sanitizers where they would be easily seen and accessible (Thaler & Sunstein, 2008; Van Bavel et al., 2020).

It is also important that individuals fully understand the consequences of applying (or not) the health behaviors, as demonstrated by the attitude component of the TPB. Therefore, governmental communications need to focus on clarifying the beneficial impact of the recommended behaviors, which will then increase positive attitudes towards them. Moreover, communication also needs to target the social norms related to the recommended behaviors. Both social approval by the community and modeling carried out by central ingroup members (e.g., “influencers” on social media that model the behavior to apply) can increase adherence to behaviors such as limitation of social contacts (Van Bavel et al., 2020). Governments also need to maintain a clear and transparent communication with the general public (Rigotti et al., 2020). Finally, our study emphasizes the fact that, when faced with a pandemic, different strategies need to be considered in order to increase adherence to the specific health behaviors as they are not associated with the same variables as everyday health behaviors.

### Limits and future perspectives

There are some limits to this study. First, due to time constraints (we attempted to collect data as early as possible when lockdown measures were introduced in Belgium) and a will to include as many predictors as possible, our items were not always selected in an optimal way to ensure good validity of all measures. The survey also only included self-reported measures, which can be subject to social desirability biases. In addition, since we decided to only investigate main effects of the TPB’s dimensions, the intention variable has not been articulated as a mediator of other dimensions as it is in the original model. Some categories of items used in the regression, such as emotional dimensions or social dimensions were defined from a factor analytic strategy based on the present data. Further use of these dimensions should involve a replication of these specific factor structures. In addition, future studies should attempt to correct the poor validity index of certain categories by improving measuring instruments (regarding the *enthusiastic/happy* emotional dimension, for instance). Furthermore, we limited our study to two health behaviors, but many others have been recommended by Belgian authorities as well as other international organizations such as WHO during the SARS-CoV-2 outbreak (e.g., wearing a facemask, coughing or sneezing in a disposable tissue or in one’s elbow, maintaining physical distance; West et al., 2020). It is worth mentioning that all significant predictors included in this study might only be relevant when assessing the application of the two behaviors we investigated. Another limitation of our study relates to its generalizability potential as we had a majority of women and individuals with a high level of education. Regarding the statistical analyses and results, we only examined main effects without looking at interactions (such as interactions effects between emotional states).

Several methodological recommendations can be made based on our research. Future studies should investigate additional variables in order to have a better comprehension of factors influencing adherence to health behaviors in the context of pandemics. One first limitation of our model relies on the absence of habits change evaluation (Orbell & Verplanken, 2020). It would have consisted in asking the extent of change between past behaviors and those currently displayed. Moreover, in their Integrated Social Cognition model, Hagger et al. (2020) suggest the inclusion of moral norms in addition to the social norms considered in the TPB to explain the application of social distancing during the SARS-CoV-2 pandemic. It is worth noticing that their dependent variable is different from the ones we considered. Also, whereas TPB presents intentions as a unique major determinant of behavior application, the Integrated Behavior Change model divides this dimension in two motivational phases (Hagger & Chatzisarantis, 2014). The first motivational phase is the cognitive formation of intentions. The second phase, namely volitional motivation, refers to a more applied representation of this intention, involving planning processes in order to actually perform the behavior intended. While the first motivational phase was examined through our intention measure, future studies should investigate in more depth the volition part of motivation, in order to better assess health behaviors application. Another approach theorized by Ajzen & Kruglanski (2019) suggests that the intention to perform a specific behavior depends on a situationally activated set of goals. For instance, regarding the limitation of social contacts, two distinct goals can be established. On the one hand, one may respect the recommendation to avoid the related fine. Another goal may rely on the will to stop the spreading of the virus. Both would concur to the implementation of the behavior, but the first might lead to less adherence to it. Future studies could integrate these dimensions to examine whether they increase the prediction of health behaviors.

The Health Action Process Approach [HAPA] (Schwarzer, 2008) also highlights factors that should be considered in order to comprehend how health behaviors can be maintained. Long-term maintenance of health behaviors in and after the SARS-CoV-2 Outbreak, such as being ready to apply certain behaviors such as physical distancing in case of a new outbreak, is a central issue. Some essential factors that should be investigated in future studies according to the HAPA model include different dimensions of self-efficacy or risk perception.

Furthermore, several predictors beyond health behavior change cognitive models could be relevant to include in further studies. For instance, obsessive-compulsive trait seems to be an interesting dimension to explore when assessing handwashing frequency (Davide et al., 2020).

Lastly, experimental designs are also necessary to study causal relationships between certain predictors and the correct application of behaviors. Overall, we believe that future studies should focus on the replication of our findings while taking into consideration the limitations mentioned above.

## Conclusion

This study provides important considerations on a large number of factors that could be associated to the adherence to behaviors recommended to adopt when faced with a pandemic. Sociodemographic variables, cognitive dimensions, and specific emotional states were central predictors to consider when examining the application of two health behaviors: frequency of handwashing and limitation of social contacts.

## Footnotes

It is important to note that different measures of appraisal were considered in our study and included in our questionnaire. Unfortunately, factorial analyses were unable to extract clear appraisals that could correspond to existing dimensions. We therefore decided to leave the measures of appraisal aside and not include them in our statistical analyses.

For exploratory reasons, items of the Individualism and Collectivism scale were added to the survey. Factorial analyses extracted new components mixing up items of the IRI and Individualism and Collectivism scales. We therefore created a composite score of empathy, that was used in the final analyses.

Three additional items regarding social relationships with colleagues, classmates and members of organizations or groups were excluded from the analyses because of the high number of participants who stated that the questions did not apply to them.

Before running the logistic regressions, we examined the potential effect of data collection time. We found some sociodemographic differences across the four weeks of data collection, but these were likely due to the different recruitment methods used than a true effect of time. For the other predictors, we found significant differences for most of them. However, the effect size was always very small (all *η*^2^ < 0.02), which indicated a relatively good stability across time. Time was therefore not included as a predictor in the logistic regressions.

Logistic regressions using several STEPWISE methods were also conducted in order to ensure the stability of our significant predictors. Results showed a perfect replication of our predictors in the first three blocks of predictors (sociodemographic, TPB, emotions) for both behaviors.

Only main effects were investigated through our analyses. Interactions were not included because of the difficulty of computing and interpreting them in a nonlinear model. Our goal was to extract the most relevant variables that are directly related to health behaviors in times of pandemics. One further reason to exclude interactions was that there is great uncertainty with regard to interpretation of interactions in logistic regressions (Norton, Wang & Ai, 2004).
